# Correlation of HBV DNA and Hepatitis B Surface Antigen Levels With Tumor Response, Liver Function and Immunological Indicators in Liver Cancer Patients With HBV Infection Undergoing PD-1 Inhibition Combinational Therapy

**DOI:** 10.3389/fimmu.2022.892618

**Published:** 2022-05-25

**Authors:** Shida Pan, Yingying Yu, Siyu Wang, Bo Tu, Yingjuan Shen, Qin Qiu, Xiaomeng Liu, Nan Su, Yanmei Zuo, Junqing Luan, Ji−Yuan Zhang, Ming Shi, Fanping Meng, Fu-Sheng Wang

**Affiliations:** ^1^ Chinese People's Liberation Army (PLA) Medical School, Beijing, China; ^2^ Department of Infectious Diseases, The Fifth Medical Center of Chinese People's Liberation Army (PLA) General Hospital, Beijing, China; ^3^ Peking University 302 Clinical Medical School, Beijing, China; ^4^ The Second School of Clinical Medicine, Southern Medical University, Guangzhou, China

**Keywords:** hepatitis B virus, hepatitis B surface antigen, HBV reactivation, median survival time, C-reactive protein, interleukin-6

## Abstract

**Background:**

Thus far, few studies have investigated the safety and efficacy of programmed death-1 (PD-1) immune checkpoint inhibitors (ICIs) and tyrosine kinase inhibitors (TKIs) antibodies in patients with hepatitis B virus (HBV)-related liver cancer.

**Objective:**

To investigate the effect of combination therapy with programmed death-1 (PD-1) immune checkpoint inhibitors (ICIs) and tyrosine kinase inhibitors (TKIs) on HBV-related liver cancer.

**Methods:**

Until January 31, 2022, liver cancer patients with hepatitis B surface antigen (HBsAg) or HBV DNA positivity, treated with PD-1 ICIs and TKIs combined with nucleoside analogs (NAs), were retrospectively reviewed. The correlation between the change in HBV DNA and HBsAg levels and tumor response was analyzed using the χ^2^ test. Cox univariate and multivariate survival analyses and Kaplan–Meier curves were used to identify and compare risk factors and overall survival (OS).

**Results:**

A total of 48 patients were enrolled in the study, with an objective response rate (ORR) of 31.3%, a disease control rate (DCR) of 66.7%; the incidence of adverse events was mostly mild. A significant decrease in HBV DNA and HBsAg levels was observed at 12 and 24 weeks compared with the baseline (p < 0.05). Compared to patients with progressive disease (PD), patients with disease control showed a more significant decrease in HBV DNA and HBsAg levels at 12 and 24 weeks (p < 0.001). Eleven patients showed elevations in HBV DNA level and one of them showed HBV reactivation; however, the reactivation was not associated hepatitis. Moreover, eight patients showed elevation in HBsAg. Elevation in HBV DNA level was associated with poor tumor response (P=0.001, OR=18.643 [95% CI: 3.271–106.253]). Cox survival analysis suggested that HBV DNA increase (P=0.011, HR=4.816, 95% CI: 1.439–16.117) and HBsAg increase (P=0.022, HR=4.161, 95% CI: 1.224–16.144) were independent risk factors associated with survival time. Kaplan–Meier curves suggested that patients who exhibited an increase in HBV DNA (6.87 months *vs* undefined, log-rank test: p= 0.004) and HBsAg (8.07 months *vs* undefined, log-rank test: p= 0.004) levels had a shorter median survival time (MST). Patients without increased HBsAg showed better baseline liver function and routine blood tests (p<0.05) than patients with increased HBsAg. An increase in C-reactive protein (CRP) and interleukin-6 (IL-6), and a decrease in T lymphocytes, CD4+ T lymphocytes, and B lymphocytes at 1-week post-treatment associated with HBsAg well-controlled.

**Conclusion:**

HBV-related liver cancer patients treated with combination therapy showed improved efficacy and safety profiles. Combination therapy has some effect on HBV infection, and a correlation between tumor response and antiviral efficacy was found. Elevation of HBV DNA and HBsAg levels may indicate poorer tumor response and survival time. Better baseline liver function and early immune activation may be associated with decline in HBsAg levels.

## Introduction

According to a status report on the cancer burden worldwide, published by the International Agency for Research on Cancer (IARC), liver cancer was predicted to be the sixth most commonly diagnosed cancer and the fourth leading cause of cancer-related deaths worldwide in 2018, with approximately 840,000 new cases and 780,000 deaths per year (1). However, more than half of the new cases and deaths occurred in China (2). Until 2017, liver cancer was the sixth most commonly diagnosed cancer and the fourth leading cause of cancer in China (3).The common causes of liver cancer include hepatitis B virus (HBV) and hepatitis C virus (HCV) infection, aflatoxin contamination, alcohol abuse, obesity, smoking, and type 2 diabetes (4). Common causes of liver cancer vary with region. In developed countries, HCV infection and alcohol abuse contribute the most to liver cancer; in China, the key etiology is chronic HBV infection, with nearly 85% of liver cancer patients having previous or current HBV infection (5). The 5-year survival rate of liver cancer patients worldwide is approximately 18%, while that in China is only 12.1% (6, 7). Most liver cancers are always detected in the middle or late stages, resulting in a poor prognosis.

Currently, tyrosine kinase inhibitors (TKIs) such as sorafenib and lenvatinib are recommended as first-line treatments for advanced liver cancer (8, 9). However, based on the existing studies, the survival benefits of sorafenib administration are limited (10, 11). Immune checkpoint inhibitors (ICIs), such as PD-1 and PD-L1, provide a new option for the treatment of patients with liver cancer. Several ICIs, such as nivolumab, pembrolizumab, and camrelizumab, have been approved for monotherapy in liver cancer cases due to the promising results in clinical trials (12–14). Although phase I/II trials have shown good efficacy and safety, phase III trials were less favorable. Although some patients have durable response to monotherapy, the benefit remains low, with an objective response rate (ORR) ranging from 15% to 20%. Therefore, combination therapies have been developed to improve ORR. Commonly used combination therapies include ICIs, targeted drugs, radiotherapy, chemotherapy, and interventional therapies (15–19). The efficacy and safety of the combination of atezolizumab and bevacizumab used in phase III IMbrave150 trial were higher compared to those obtained with sorafenib, with the results indicating a higher 12-month survival rate (67.2% *vs*. 54.6%) and median progression-free survival (PFS) (6.8 months *vs* 4.3 months); however, the incidence of immune-related adverse events (irAEs) was similar (20).

Although ICI treatment has shown better efficacy than sorafenib in liver cancer, there are few reports on its safety and efficacy in patients with HBV-associated liver cancer, especially in those with high viral loads. This is due, firstly, to the HBV viral load that is associated with carcinoma recurrence and prognosis. Patients with HBV DNA > 10^5^ IU/mL before transplantation showed frequent HCC (hepatocellular carcinoma) recurrence after transplantation (21). Patients with a HBV DNA load of < 2000 IU/mL have longer recurrence-free survival (RFS) rate, and a complete virologic response after operative therapy is associated with a lower risk of cancer recurrence (22). High serum levels of HBV DNA before transarterial chemoembolization (TACE) are associated with poor overall survival (OS) and rapid progression of HCC after TACE (23). Except surgical treatment, the HBV viral load was also associated with poor prognosis in patients receiving systemic therapy. Poor OS was recorded for patients with detectable HBV DNA levels after antiangiogenic therapy; the baseline HBV DNA level was an independent predictor of poor OS (24). High baseline HBV load was an adverse prognostic factor for survival in patients treated with sorafenib (25). A high viral load before treatment was an exposure risk factor for survival during systemic chemotherapy and was associated with a higher incidence of severe hepatitis during treatment (26). In most clinical trials for PD-1/PD-L1 ICIs, liver cancer patients with HBV infection were required to have a baseline HBV load <100 IU/mL for eligibility; therefore, the effect of baseline viral load on clinical outcomes could not be assessed. However, several studies have enrolled patients with a high viral load, suggesting an absence of correlation between baseline HBV viral load and patient OS and PFS during PD-1/PD-L1 treatment; however, the presence or absence of antiviral therapy during immunotherapy could affect tumor prognosis (27, 28).

Secondly, HBV reactivation could affect the use of PD-1/PD-L1 ICIs in HBV-related liver cancer patients. In HBV-related liver cancer patients, common triggers for HBV reactivation include surgery, radiation therapy, chemotherapy, and immunosuppressive drugs. For example, in patients with HBV reactivation and HBV reactivation-associated hepatitis after chemotherapy, use of prophylactic antiviral drugs were independent risk factors for HBV reactivation (29, 30). In patients treated with partial hepatectomy, radiofrequency ablation, and interventional therapy, HBV reactivation and HBV reactivation-related hepatitis may be associated with the immunosuppressive state caused by surgery and anesthesia as well as surgical injury of the liver (31–33). HBV reactivation has also been reported in PD-1PD-L1 antibody therapy. In an Asian cohort of CheckMate 040, patients were subjected to antiviral therapy to achieve an HBV DNA load of ≤ 100 IU/mL; however, five patients exhibited an increase of > 1log_10_ IU/mL of HBV DNA after PD-1 ICI immunotherapy but did not develop hepatitis (34). Studies that included patients with an HBV DNA load of >100 IU/mL found that the baseline viral load had no effect on HBV reactivation, but antiviral therapy was an independent risk factor for HBV reactivation (35, 36).

Although PD-1PD-L1 ICIs exhibit good efficacy and safety in patients with HBV-related liver cancer, there are a few shortcomings that need to be addressed. For example, the load of HBV in patients enrolled in studies is usually low (< 2000 IU/mL), and the efficacy and safety of the drugs against high viral loads (> 2000 IU/mL) remains unclear. For patients with high viral loads, is it necessary to keep the viral load level low before initiating PD-1/PD-L1 antibody immunotherapy? Does the presence of HBV re-replication or reactivation indicate a poor prognosis for patients compared with those with a decrease in HBV DNA? To address these questions, further studies are required.

For patients with HBV-related liver cancer, PD-1/PD-L1 antibodies not only showed good tumor response efficacy but also played a role in antiviral treatment of HBV infection (37, 38). In some pre-clinical studies, PD-1 was highly expressed on HBV-specific CD8+ T cells in virally persistently infected mice than in HBsAg-clear mice; PD-1 antibody treatment restored the function of HBV-specific CD8+ T cells and promoted HBsAg clearance (39–41). Nivolumab, with and without therapeutic vaccination, when used on serum-positive chronic hepatitis B (CHB) patients, resulted in HBsAg reduction and clearance in some patients (42). Although PD-1/PD-L1 ICIs are effective in both tumor control and HBV infection, no study has investigated whether there is an association between tumor response and viral reduction. It is also unknown whether baseline characteristics and dynamics of clinical parameters during PD-1/PD-L1 treatment are associated with decrease HBsAg levels.

In our study, liver cancer patients did not need to maintain lower HBV DNA levels when subjected to combined treatment with PD-1 ICIs and TKIs, but concurrent initiation of nucleoside analog (NA) antiviral therapy was required. The aim of the study is to investigate the efficacy, safety (incidence of adverse events and hepatitis related to HBV reactivation) and impact on HBV DNA and HBsAg levels of combination therapy, and to determine the correlation between HBV replication or reactivation with patient response and survival.

## Materials and Methods

### Patients’ Inclusion

Between March 2020 and January 2022, patients diagnosed with liver cancer through histological or imaging examination, with HBV DNA or HBsAg seropositivity and treated with at least one cycle of PD-1 ICIs combined with TKIs, were retrospectively reviewed. All patients were treated in the Department of Infectious Diseases, Fifth Medical Center of PLA General Hospital. Sintilimab (Innovent Biologics and Eli Lilly and Company) and camrelizumab (Jiangsu Hengrui Medicine Co. Ltd.), at a fixed dose of 200 mg, and toripalimab at 240 mg were prescribed for a three week cycle. Sorafenib (Bayer Schering Pharma AG) was administered at 400 mg/day, lenvatinib (Eisai Co. Ltd) at 8 to 12 mg/day and regorafenib (Bayer) at 80 mg/day, according to body weight. Treatment options for patients who met the criteria for TACE treatment were decided together by the patient and the physician before or after systemic combination therapies. NAs including entecavir (ETV) (0.5 mg/day), tenofovir (TDF) (300 mg/day), and adefovir dipivoxil (ADV) (10 mg/day) were provided simultaneously with immunotherapy.

Radiological response was recorded using computed tomography (CT) or magnetic resonance imaging (MRI) initially as the baseline and then at 3 month intervals. Tumor response was assessed by the modified Response Evaluation Criteria in Solid Tumors (mRECIST) (43, 44). Taking the baseline sum of the diameters of target lesions as a reference, complete response (CR) was defined as the disappearance of any intratumoral arterial enhancement in all target lesions; at least a 30% decrease in the sum of diameters of target lesions was defined as a partial response (PR); an increase in the sum of the diameters of at least 20% was defined as progressive disease (PD); and no change in the sum of the diameters between PR and PD was defined as stable disease (SD). IrAEs were documented according to Version 5 of the Common Terminology Criteria for Adverse Events (CTCAE) (45). Patients with spontaneous bacterial peritonitis, a complication of primary liver cancer, were excluded from this study. Depending on the toxicity grade, TKI dose was reduced, suspended, discontinued, or switched to other TKIs in patients who developed irAEs associated with TKI treatment. PD-1 ICIs were suspended or discontinued, and immunosuppressant agents were administered based on the severity of irAEs.

HBV reactivation was defined according to the American Association for the Study of Liver Diseases (AASLD) hepatitis B guidance in 2018 for HBsAg-positive and anti-hepatitis B core (HBc)-positive patients, including (1) an increase in HBV DNA ≥ 2 log (100-fold) compared to the baseline level, (2) HBV DNA ≥ 3 log (1000) IU/mL in a patient with previously undetectable levels, or (3) HBV DNA ≥ 4 log (10,000) IU/mL if the baseline level was not available. HBsAg-negative and anti-HBc-positive patients who met the following criteria were considered to have HBV reactivation hepatitis: detectable HBV DNA or reverse serological conversion of HBsAg or HBV reactivation accompanied with an increase in alanine aminotransferase (ALT) ≥ 3 times the baseline level and absolute value > 100 U/L (46).

The cut-off date for follow-up was January 31, 2022, and all data were obtained from patient medical records. The baseline data included patient demographics, such as Child-Pugh stage, Barcelona Clinic Liver Cancer (BCLC) stage, serum alpha-fetoprotein (AFP) level, type of combination therapy, and TACE treatment. The data pertaining to the levels of HBV DNA and HBsAg, routine blood tests, liver function, lymphocyte subsets, C-reactive protein (CRP), and interleukin-6 (IL-6) at baseline and after 1 week were collected. HBV DNA was quantified using real-time fluorescence PCR (Roche COBAS, USA) with a lower limit of 20 IU/mL. HBsAg was quantified using a Beckman AU5800-03 with a lower limit of 0.05 IU/ml. Serum CRP and liver function parameters were measured using an automatic biochemical analyzer (AU5400; Beckman Coulter, Brea, CA), with the upper limit of the normal value for serum CRP set at 8.2 mg/L. Serum IL-6 was examined using a Roche Cobas 8000 (Roche Diagnostics GmbH, Mannheim, Germany), and the upper limit of the normal value for serum IL-6 was set at 7 pg/mL. Lymphocyte subsets and counts were measured using a FACSCalibur flow cytometer (BD Biosciences, Becton, NJ). Routine blood parameters were measured using an automatic hematology analyzer (HN-2000 series; SYSMEX, Kobe, Japan).

### Statistical Analysis

All statistical analysis were conducted using the IBM SPSS Statistics software application (version 25.0; IBM, Armonk, NY, USA). The baseline data and adverse events were summarized using descriptive statistics. HBV DNA and HBsAg levels below the lower limit were recorded as the lower limit of detection. Continuous variables that conformed to a normal distribution were represented as mean ± SD, non-normal distribution data were presented using median (quartiles), and categorical data by variable numbers (percentages). The student t test was used to compare continuous variables that conformed to a normal distribution. The χ^2^ test or Fisher’s exact test were used to compare categorical data. Comparisons between the two groups were performed using the nonparametric Mann–Whitney U test. Comparisons within groups were analyzed using nonparametric Wilcoxon’s paired test. The cut-off date for survival analysis was January 31, 2022, and OS was estimated using Kaplan–Meier curves and compared using the log-rank test. Hazard ratios (HRs) and 95% confidence intervals (CIs) were analyzed using Cox univariate and multifactorial survival analyses to identify any independent predictive factors that were associated with OS. All figures were generated using GraphPad Prism statistical software (version 9.0; GraphPad Software, San Diego, CA, USA). Differences were considered statistically significant at p < 0.05.

## Results

### Baseline Characteristics of Patients

A total of 48 patients were enrolled in this study: 41 with HCC and 7 with cholangiocarcinoma. Thirty-four patients were treated with a combination of sintilimab and lenvatinib, while 14 patients received other combination therapies. Twelve patients underwent TACE during the treatment period. Thirty-three patients had received antiviral therapy prior to combination therapy and 15 patients had never received antiviral therapy. Fifteen patients had baseline HBV DNA levels below the lower limit of detection, 16 patients had HBV DNA levels >1000 IU/mL, and other patients had HBV DNA levels between 20 IU/mL and 1000 IU/mL. The baseline HBsAg level of five patients was not available; one patient was seronegative for baseline HBsAg but with HBV DNA > 20 IU/mL, and 45 patients had HBsAg levels > 0.5 IU/mL. Other details are listed in **Table 1**.

**Table 1:** Baseline characteristics of patients enrolled in the study.

	n = 48
Age (year)	55.96 ± 9.72
Sex	
Male	41 (85.4%)
Female	7 (14.6%)
Types of tumors	
hepatocellular carcinoma	43 (89.6%)
Cholangiocarcinoma	5 (10.4%)
BCLC stage	
B	12 (25.0%)
C (PVTT)	22 (45.8%)
C (M)	14 (29.2%)
Child-Pugh stage	
A	26 (54.2%)
B	22 (45.8%)
AFP	
<400 (IU/ml)	27 (56.2%)
≥400 (IU/ml)	21 (43.8%)
Previous antiviral therapy	
Yes	33 (68.7%)
No	15 (31.3%)
HBV DNA	
≤20 (IU/ml)	15 (31.2%)
>20 and ≤10^3 (IU/ml)	17 (34.5%)
>10^3 (IU/ml)	16 (33.3%)
HBsAg	
≤0.05 (IU/ml)	1 (2%)
>0.05 (IU/ml)	42 (87.6%)
No available	5 (10.4%)
HBeAg state	
Seropositive	9 (18.8%)
Seronegative	38 (79.2%)
No available	1 (2%)
Combination treatment	
Sintilimab+Lenvatinib	34 (70.8%)
Sintilimab+Sorafenib	7 (14.6%)
Camrelizumab+Lenvatinib	3 (6.3%)
Toripalimab+Lenvatinib	4 (8.3%)
Combinated TACE therapy	
Yes	12 (25.0%)
No	36 (75%)
Combination treatment as systemic	
First line	21 (43.8%)
Second line	5 (10.4%)
Third line	16 (33.3%)
Fourth line	6 (12.5%)
Antiviral therapy	
ETV	38 (79.2%)
ADV	1 (2%)
TDF	4 (8.3%)
ETV+TDF	4 (8.3%)
Tumor response	
CR	2 (4.2%)
PR	13 (27.1%)
SD	17 (35.4%)
PD	16 (33.3%)
OR (CR+PR)	15 (31.3%)
DC (CR+PR+SD)	32 (66.7%)

ADV, Adefovir dipivoxil; AFP, Alpha‐Fetoprotein; BCLC, Barcelona Clinic Liver Cancer; CR, complete response; DC, disease control; ETV, Entecavir; HBeAg, hepatitis B e antigen; HBsAg, hepatitis B surface antigen; HBV, hepatitis B viral; OR, objective response; PD progressive disease; PR, partial response; SD, stable disease; TACE, transcatheter arterial chemoembolization; TDF, Tenofovir.

### Immune-Related Adverse Events and Tumor Response Profiles

According to mRECIST, two (4.2%) patients had CR, 13 (27.1%) had PR, 17 (35.4%) had SD, and 16 (33.3%) had PD, with an ORR of 31.3% and Disease control rate (DCR) of 66.7%, as shown in **Table 1**.

There were 22 (45.8%) patients who experienced at least one adverse event of any grade, and 12 patients (25%) developed grade 3 (G3)/grade 4 (G4) irAEs. The most common adverse events in G3/G4 irAEs were fever (n = 9, 18.8%), fatigue (n = 7, 14.6%), lymphopenia (n = 2, 4.2%), bacterial infection (n = 2, 4.2%), diarrhea (n = 2, 4.2%), and hepatitis (n = 2, 4.2%). All patients who experienced severe irAEs improved after receiving glucocorticoids according to clinical guidelines. The overall occurrence of adverse events in the patients is shown in **Table 2**.

**Table 2:** Occurrence of adverse events.

	G1/G2	G3/4
Fever	9 (18.8%)	0
Fatigue	7 (14.6%)	0
Hypertension	6 (12.5%)	0
Diarrhea	6 (12.5%)	2 (4.2%)
Rash	6 (12.5%)	0
Hypothyroidism	4 (8.3%)	0
Pruritus	2 (4.2%)	0
Proteinuria	3 (6.3%)	0
Renal dysfunction	2 (4.2%)	0
Pneumonia	1 (2%)	1 (2%)
Lymphopenia	1 (2%)	2 (4.2%)
Thrombocytopenia	1 (2.1%)	1 (2.1%)
Hyperthyroidism	1 (2.1%)	0
Cardiotoxicity	1 (2.1%)	0
hoarseness	1 (2.1%)	0
hepatitis	0	2 (4.2%)
Bacterial infection	0	2 (4.2%)
fungal infection	0	1 (2.1%)
Herpes virus infection	0	1 (2.1%)
Intestinal infections	0	1 (2.1%)

During treatment, four patients permanently discontinued sintilimab, three due to irAEs, and one due to tumor progression; five patients had their TKI dose reduced due to adverse AEs; six patients discontinued TKIs, two due to AEs, and four due to complications such as upper gastrointestinal bleeding. Twelve patients switched to regorafenib, three due to AEs, and nine due to tumor progression.

### Antiviral Efficacy of Combination Therapy

**Figure 1:**
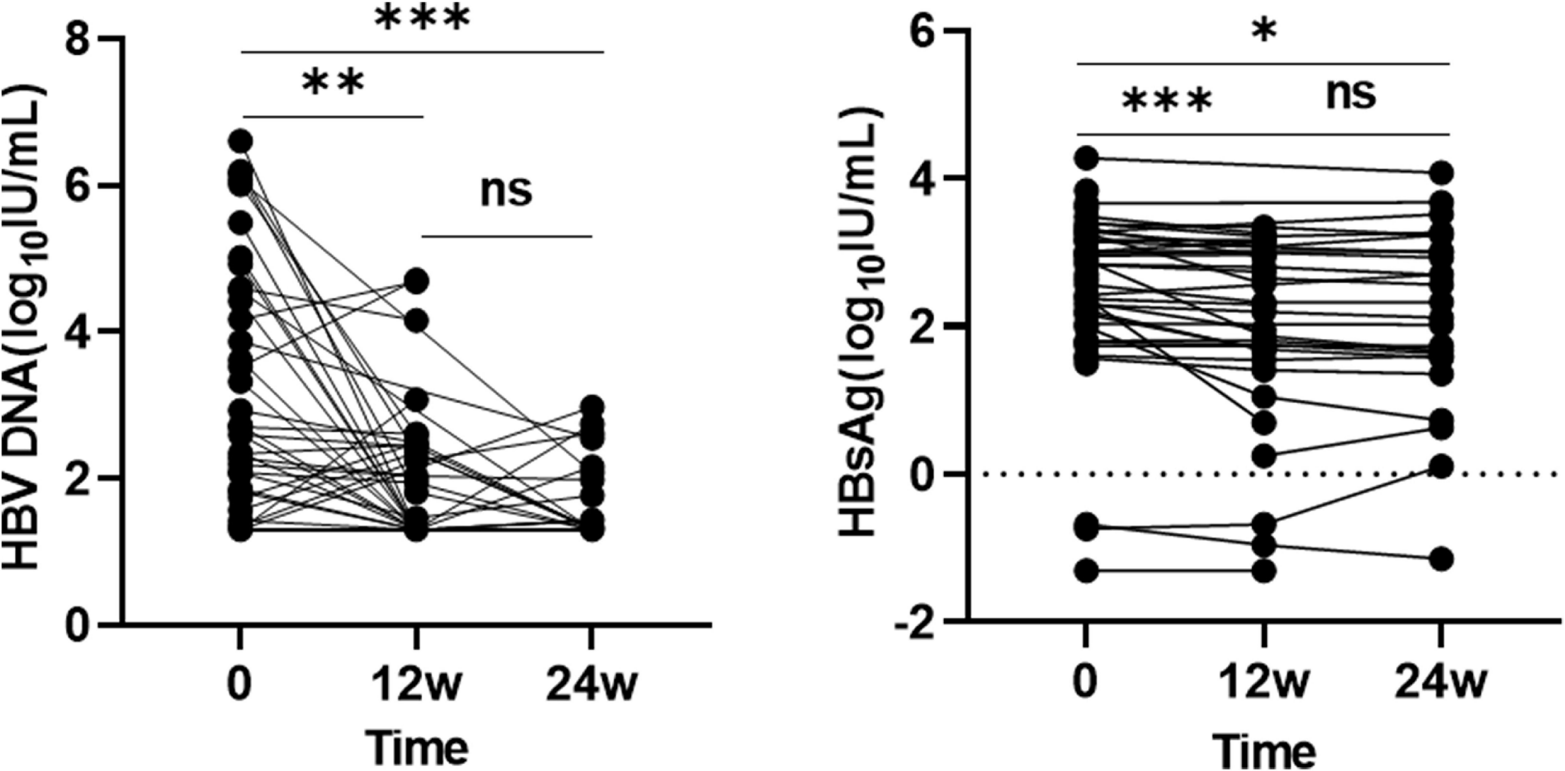
The overall changes in HBV DNA and HBsAg levels at different time point. ns: >0.05; ***: ≤0.005; **: ≤0.01; *: ≤0.05.

Patients were treated with a combination therapy of PD-1 ICIs and TKIs. All patients received antiviral therapy with NAs and were followed up for more than 24 weeks. One patient was HBsAg negative and HBV DNA positive at the start of treatment. One patient died within 12 weeks. The follow-up time of 12 patients was more than 12 weeks but not more than 24 weeks, during which time, seven patients died, two were lost to follow-up, and three were still subject to follow-up. Fifteen patients had baseline HBV DNA levels below the lower limit of detection, of which three patients had positive HBV DNA after 24 weeks of treatment, and one of three patients reached HBV reactivation. Twelve patients with baseline HBV DNA positivity had HBV DNA clearance at 12 weeks and five at 24 weeks. HBsAg changes could not be assessed in nine patients at 12 or 24 weeks due to lack of baseline data. Compared with the established baseline levels, overall HBV DNA and HBsAg levels showed a statistically significant difference at 12 and 24 weeks (p < 0.05) (**Figure 1**).

**Figure 2:**
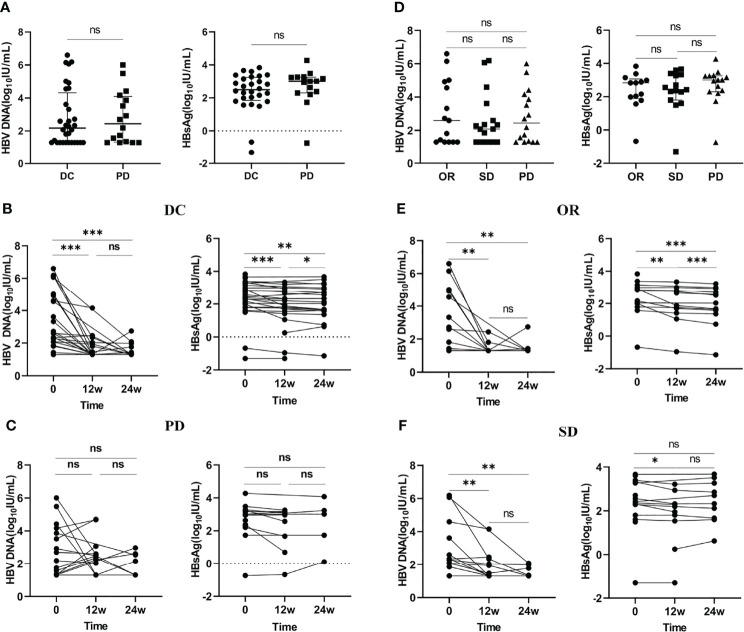
The change in HBV DNA and HBsAg levels in patients with different tumor responses. **(A)** Comparison of baseline levels of HBV DNA and HBsAg between patients with DC and PD. **(B)** The changes in HBV DNA and HBsAg levels at different time points in patients with DC. **(C)** The changes in HBV DNA and HBsAg levels at different time points in patients with PD. **(D)** Comparison of baseline levels of HBV DNA and HBsAg among patients with OR, SD and PD. **(E)** The changes in HBV DNA and HBsAg levels at different time points in patients with OR. **(F)** The changes in HBV DNA and HBsAg levels at different time points in patients with SD. DC, disease control; OR, objective response; PD, progressive disease; SD, stable disease. ns: >0.05; ***: ≤0.005; **: ≤0.01; *: ≤0.05.

To investigate the correlation between patient tumor response and virus control, we analyzed the changes in HBV DNA and HBsAg levels in patients with different tumor responses. The results suggested that there was no statistically significant difference in HBV DNA and HBsAg levels between patients with tumor control (including CR, PR, SD) and tumor progression (PD), and no significant difference was observed among patients with CR plus PR, SD, and PD (p > 0.05) (**Figures 2A, D**). Compared to patients with PD, patients with tumor control showed a significant decline in HBV DNA and HBsAg levels at baseline, 12 weeks, and 24 weeks (p<0.05) (**Figures 2B, C**). In addition, we divided patients with DCR into CR plus PR and SD groups and analyzed the changes in HBV DNA and HBsAg levels in the two groups. The results showed that patients with CR plus PR and SD had similar changes in HBV DNA levels at baseline, 12 weeks, and 24 weeks. However, the change in HBsAg levels was more significantly different in CR plus PR patients than in SD patients (**Figures 2E, F**).

### Correlation Between Elevated HBV DNA, HBsAg Levels, Tumor Response, and Survival Time

After 24 weeks of treatment, 11 patients had elevated HBV DNA and ALT or aspartate aminotransferase (AST) levels, one of whom had HBV reactivation; however, this patient did not develop HBV reactivation-related hepatitis. Two patients developed hepatitis, but it was not associated with HBV reactivation. The characteristics of the 11 patients with elevated HBV DNA levels are shown in **Table S1**. In addition, 8 patients suffered from elevated HBsAg levels, the characteristics of which are shown in **Table S2**.

Nine of the 11 patients who suffered from HBV DNA elevation had PD, one patient had SD, and one patient had PR. Four of the eight patients with elevated HBsAg levels had SD and four patients had PD. The results of the χ^2^ test suggested that HBV DNA elevation was significantly associated with poor tumor response (p=0.001, OR=18.643 [95% CI: 3.271–106.253]), but elevated HBsAg was not associated with tumor response (p=0.270, OR=2.440 [95% CI: 0.499–11.965]) (**Table 3**).

**Table 3:** χ^2^ test for change trends of HBV DNA and HBsAg levels according to tumor response.

	CR+PR+SD	PD	p value	OR
The change of HBV DNA			<0.001	18.643 (3.271-106.253)
Without increase	29	7		
With increase	2	9		
The change of HBsAg			0.270	2.44 (0.499-11.965)
Without increase	22	9		
With increase	4	4		

AFP, Alpha‐Fetoprotein; BCLC, Barcelona Clinic Liver Cancer; HBsAg, hepatitis B surface antigen; HBV, hepatitis B viral; HBsAg, hepatitis B surface antigen; MST, median survival time; TACE, transcatheter arterial chemoembolization.

**Figure 3:**
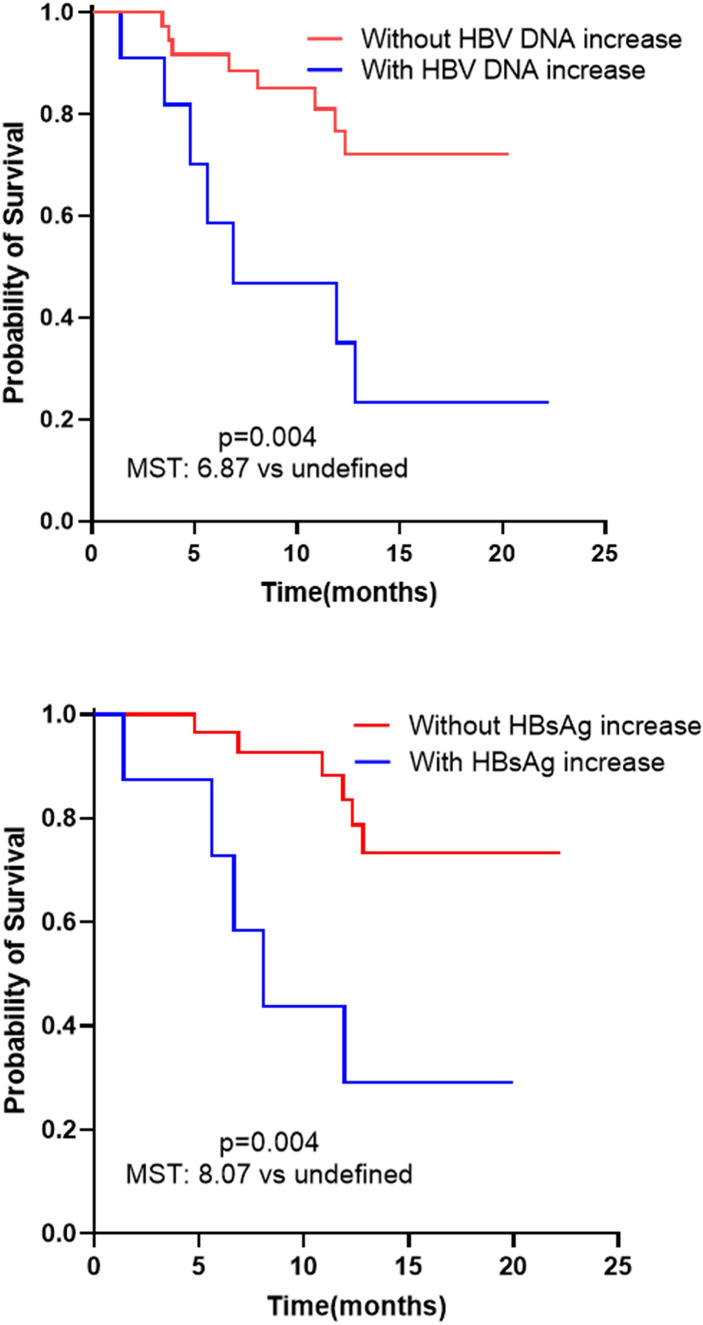
Correlation analysis between tumor response, survival time and the elevation of HBV DNA and HBsAg levels. Kaplan–Meier overall survival estimate curves in patients with increasing HBV DNA and HBsAg and patients exhibiting no increase in HBV DNA and HBsAg. HBsAg, hepatitis B surface antigen; HBV, hepatitis B viral; MST, median survival time.

The results of Cox univariate and multivariate survival analyses suggested that increase in HBV DNA (P=0.011, HR=4.816, 95 CI%: 1.439–16.117) and HBsAg (P=0.022, HR=4.161, 95 CI%: 1.224–16.144) levels were independent risk factors associated with patient survival time (**Table 4**). Patients who had increased HBV DNA (6.87 months *vs* undefined, log-rank test: p= 0.004) and HBsAg (8.07 months *vs* undefined, log-rank test: p= 0.004) levels had a shorter median survival time (MST) than patients without an increase in HBV DNA and HBsAg levels (**Figure 3**).

**Table 4:** Cox univariate and multifactorial survival analysis to identify any independent predictive factor associated with OS.

	Cox univariate survival analysis			Cox multivariate survival analysis		
	HR	95%CI	p value	HR	95%CI	p value
Age	0.979	0.925-1.036	0.461			
Sex	0.785	0.420-1.465	0.446			
Types of tumors	1.062	0.141-8.030	0.953			
BCLC stage	1.165	0.644-2.105	0.614			
Child-Pugh stage	0.140	0.160-1.294	0.140			
Options of combination therapy	1.154	0.426-3.125	0.778			
TACE treatment	0.329	0.075-1.439	0.140			
Line of treatment	0.9103	0.579-1.440	0.696			
AFP	2.312	0.855-6.257	0.099			
The occurrence of irAEs	0.491	0.185-1.298	0.152			
Prior antiviral therapy	1.176	0.406-3.411	0.765			
Baseline level of HBV DNA	1.012	0.753-1.358	0.939			
HBV DNA increase	4.034	1.456-11.179	0.007	4.816	1.439-16.117	0.011
HBsAg increase	4.869	1.476-16.064	0.009	4.161	1.224-16.144	0.022

AFP, Alpha‐Fetoprotein; BCLC, Barcelona Clinic Liver Cancer; HBsAg, hepatitis B surface antigen; HBV, hepatitis B viral; TACE, transcatheter arterial chemoembolization.

### Correlation Between Changes in Clinical Parameters and Elevated HBsAg

**Figure 4:**
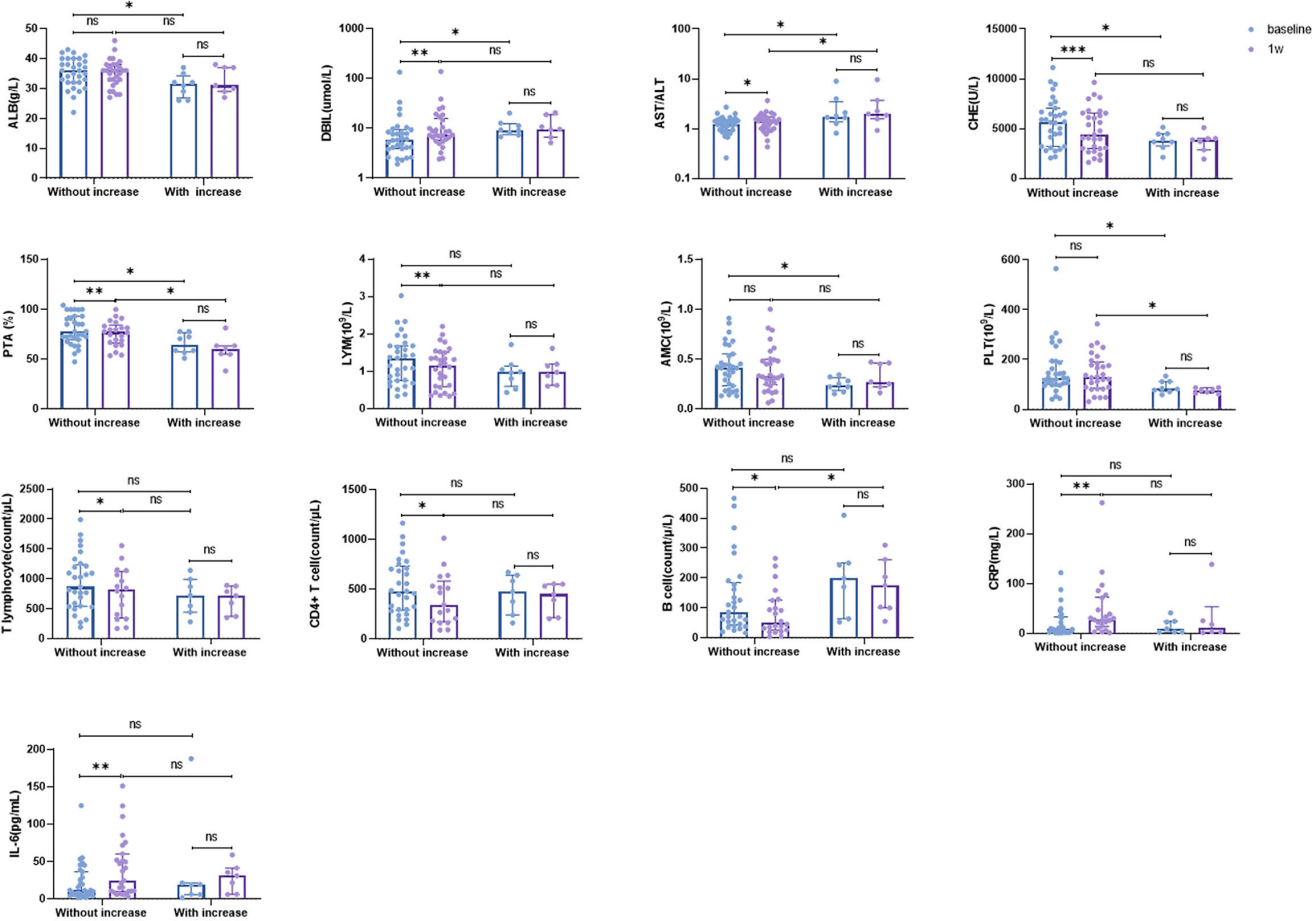
Baseline characteristics and trends of some clinical parameters in patients with or without increasing HBsAg level. ALB, albumin; AMC, absolute monocyte count; CHE, cholinesterase; CRP, C-reactive protein; DBIL, direct bilirubin; IL-6, interleukin-6; LYM, absolute lymphocytes; PLT, platelets; PTA, prothrombin activity. ns: >0.05; ***: ≤0.005; **: ≤0.01; *: ≤0.05.

To identify the biomarkers associated with elevated HBsAg levels, we analyzed the baseline characteristics and early change trends in some clinical parameters. The results suggested that there were higher baseline levels of albumin (ALB), AST/ALT ratio, cholinesterase (CHE), prothrombin activity (PTA), and platelets (PLT), and lower baseline levels of direct bilirubin (DBIL) and absolute value of monocytes (AMC) in patients without increased HBsAg than in patients with increased HBsAg. Compared to patients with increased HBsAg, other patients showed an increase in CRP, IL-6, AST/ALT ratio, and DBIL and a decrease in T lymphocytes, CD4+ T lymphocytes, B lymphocytes, and CHE one week after the initiation of treatment (**Figure 4**).

## Discussion

In our study, we investigated the safety and efficacy of a combined treatment with PD-1 ICIs and TKIs in liver cancer patients with HBV infection, the effect of combination therapy on HBV DNA and HBsAg levels, and biomarkers associated with HBsAg elevation.

Combination therapy showed good safety and efficacy; the ORR and DCR was 31.5% and 66.7%, respectively. There were 22 (45.8%) patients with irAEs, of which 10 patients suffered from grade 3/4 irAEs, but their symptoms improved upon the administration of glucocorticoids, and no deaths were associated with irAEs. Patients suggested a decrease in overall HBV DNA and HBsAg levels. Compared with PD patients, patients with DCR (including CR, PR and SD) showed a more significant decline in HBV DNA and HBsAg levels. Eleven patients showed an increase in HBV DNA level within 24 weeks; one of these patients met the criteria for HBV reactivation, but did not develop HBV-related hepatitis. The baseline level of HBV DNA between patients with and without elevated HBV DNA was not statistically different, and univariate regression analysis suggested that the baseline level of HBV DNA was not a risk factor for HBV DNA elevation. The χ^2^ test showed an association between elevated HBV DNA level and tumor response, and patients with elevated HBV DNA level were more likely to experience tumor progression. Survival analysis revealed that patients who developed elevated HBV DNA levels had a shorter survival than those who did not, with a median OS of only 6.87 months. To the end, eight patients developed elevated HBsAg levels within 24 weeks, and elevated HBsAg level showed a shorter median OS (8.07 months). Furthermore, patients with increased HBsAg level showed lower levels of ALB, CHE, PTA, monocytes, and PLT and higher levels of DBIL and AST/ALT ratio at baseline. There was a decrease in CHE, PTA, LYM, T lymphocytes, CD4+ T cells, and B cells, and an increase in DBIL, AST/ALT ratio, CRP, and IL-6 in patients without increased HBsAg one week after treatment initiation, but these results were not observed in patients with increased HBsAg.

HBV DNA is often associated with poor prognosis in the local or systemic treatment of liver cancer (21–28). However, during PD-1/PD-L1 ICI treatment, patients with HBV-related liver cancer had a similar tumor response to that of all patients. For example, in the CheckMate 040 study, the ORR of nivolumab monotherapy was 19% and 14% in all patients and the HBV subgroup, respectively, and the ORR of nivolumab and ipilimumab was 31% in both the groups (12, 15). In the KEYNOTE-224 study, the DCR was 60% and 52% in all patients and HBV infection group, respectively (13). The ORRs were 36% and 33% in patients treated with atezolizumab and bevacizumab, respectively. However, these patients started immunotherapy with a low viral load (less than 500 IU/mL), which may have contributed to the impact of HBV DNA being overlooked. In our study, 16 patients had HBV DNA levels > 1000 IU/mL, and 11 patients had HBV DNA levels less than 1000 IU/mL but more than 100 IU/mL. The overall ORR and overall DCR were 31.3% and 66.7%, respectively, most of the irAEs were mild, and no patient died from adverse events. In patients with HBV DNA levels > 100 IU/mL, one patient had CR, seven patients had PR, and ten patients had SD; the ORR and DCR were 29.6% and 66.7%, respectively. From the results of the mate analysis, the overall ORR and DCR were 20% and 60%, respectively, for PD-1/PD-L1 ICI monotherapy in liver cancer patients, while the ORR was 29% and DCR was 77% for combination therapy with anti-VEGF drugs; the overall rate of irAE was 63%, and the occurrence of severe adverse events was 11% (47). Our results suggest that regardless of the HBV DNA levels, there were similar tumor responses and irAEs between patients with HBV-associated liver cancer and other liver cancer patients. Therefore, HBV infection may not be a promoter during PD-1/PD-L1 therapy in patients with liver cancer. However, the sample size considered in this study was small; future studies require larger sample sizes to confirm this observation.

CD8+ T cells play an important role in virus clearance and are critical for controlling the progression of CHB (48–50). CD8+ T lymphocytes play an important role in tumor immunity and are also crucial in virus clearance and regulation of CHB infection (48–50). Exhaustion of CD8+ T cells could be found in CHB patients, characterized by upregulated expression of PD-1 accompanied by a reduction in secretion and killing functions. Therefore, blocking the PD-1/PD-L1 signaling pathway can restore the function of CD8+ T cells and promote virus clearance. In our study, we investigated the effect of PD-1 antibodies on HBV, and the results suggested that patients showed a decrease in overall HBV DNA and HBsAg levels when using a prophylactic antiviral. Furthermore, patients with DCR showed better efficacy of antiviral therapy and more pronounced decrease in HBV DNA and HBsAg levels compared to those with PD. We hypothesized that this may be related to the reactivation of exhausted CD8+ T cells (51). However, a recent study indicated that tumor-infiltrating HBV-specific CD8 T cells have been proven to be associated with prolonged patient relapse-free survival. More HBV-specific CD8 T cells could be detected in both liver cancer tissue and healthy tissue compared to their levels in the tissues of relapsed patients (52). This indicates that restoring the function of HBV-specific CD8 T cells may be important for improving both the tumor response and virus clearance in patients with HBV-related liver cancer. However, in our study, patients treated with both PD-1 antibodies and NAs did not show HBsAg clearance. Although the non-clearance could be related to the immunosuppressed state, it suggests that when choosing PD-1/PD-L1 antibodies for antiviral therapy, the efficacy of single-agent or combined NA therapy may not be satisfactory, and combination therapy with other treatment options should be considered to improve HBsAg clearance.

HBV reactivation is a key issue that requires close attention during the treatment of patients with HBV-infected liver cancer. HBV reactivation has been observed in both local and systemic treatments, irrespective of HBV DNA levels, and the use of prophylactic antiviral drugs is an independent risk factor for HBV reactivation (29–36). Although HBV reactivation can also occur during PD-1/PD-L1 ICI treatment, it is less frequent than during surgery, radiation therapy, and other treatments. Treatment such as surgery and radiation therapy can lead to immunosuppression accompanied by dysfunctional HBV-specific T and B cells, resulting in HBV DNA replication and HBV reactivation (53). However, PD-1/PD-L1 antibodies promote the reactivation of the immune system by blocking inhibitory signaling pathways to inhibit viral replication (41). At present, the precise mechanism of HBV re-replication or reactivation in patients treated with PD-1/PD-L1 antibodies is unknown. In a recent study, researchers reported that virological breakthrough(VB) was an indicator of poor outcome in patients with HBsAg positive HCC under ICIs therapy (54). This finding is similar with our study. In our research, all patients underwent PD-1 combined with targeted drug therapies. We found that some patients who had elevated HBV DNA did not meet the requirements for VB or HBV reactivation. We included this group of patients in the study and found similar results, expanding the applicability of our conclusions. Besides HBVDNA, most patients underwent a regular post-treatment HBsAg assay, which enabled us to assess the impact of ICIs on HBsAg. Liver function and immunological characteristics were evaluated based on the change trend of HBsAg in our study. We discussed the relationship between the changes of HBsAg and inflammatory factors, lymphocyte subsets and other immunological indicators. In our study, 11 patients showed HBV DNA elevation, resulting in HBV reactivation in one of them, but it did not lead to HBV reactivation-related hepatitis. In all patients with HBV DNA elevation, one patient had baseline HBV DNA > 10000 IU/mL, one patient had baseline HBV DNA >1000 IU/mL, two patients had baseline HBV DNA > 100 IU/mL, and other patients had HBV DNA < 100 IU/mL. Univariate regression analysis suggested that the baseline HBV DNA level was not a risk factor for HBV DNA elevation. This result suggests that there is no correlation between baseline HBV DNA levels and HBV DNA increase. Keeping viral load at a lower level when starting PD-1 therapy is therefore not necessary if antiviral therapy is also available, and a high viral load should not be a contraindication to PD-1/PD-L1 ICI therapy in patients with HBV-related liver cancer. In addition, in our study, we found that elevated HBV DNA levels were associated with tumor response and patient survival. These results suggest that elevated or reactivated HBV DNA may occur due to poor tumor control, or that elevated HBV DNA may imply poor prognosis; however, due to the small sample size, the relationship between the two remains unclear. The existence of this correlation may explain why HBV reactivation occurred during treatment with PD-1/PD-L1 ICIs, a class of immune activators. It is possible that patients with PD experience immunosuppression and lack of activated immune cells, such as depleted CD8 T cells, which is consistent with the immunosuppressed state caused by other treatments (53). However, this hypothesis and its exact mechanism require further study.

Along with an increase in HBV DNA level, we also observed elevated HBsAg levels during treatment. Up to 24 weeks, eight patients did not show a decrease in HBsAg, and patients who developed HBsAg elevation had shorter OS. Although our results did not suggest a correlation between HBsAg decline and tumor response, this may be due to missing HBsAg data after treatment in some patients, or there may not be an intrinsic correlation. The exact reasons need to be explored considering a larger sample size. Furthermore, we investigated the correlation between changes in clinical parameters and HBsAg levels. These results suggested that better baseline liver function was associated with changes in HBsAg levels. As an immune organ, the liver can mediate adaptive immune tolerance, and better liver function implies better immune regulation, which may play a role in HBsAg clearance (55). Furthermore, an early increase in CRP and IL-6 levels and a decrease in lymphocyte subsets are associated with a decline in HBsAg levels. Although the exact mechanisms responsible for the changes in immune markers are not known, there is an association between changes in CRP and IL-6 levels and changes in lymphocyte subsets. This may be related to the systemic inflammatory response caused by a cytokine storm, which has also been seen in COVID-19 patients. Compared with mild-type COVID-19 patients, severe-type patients who experienced cytokine storms had higher levels of CRP and IL-6, which are characterized by high levels of granulocyte colony stimulating factor, interferon-inducible protein-10, monocyte chemotactic Protein-1, macrophage inflammatory protein-1α/β, IL-8, and other cytokines, which can promote chemotaxis or apoptosis of peripheral blood lymphocyte subsets, leading to a decrease in cell numbers (56–59).

However, this study had several limitations. First, this was a single-center retrospective study with a relatively small sample size. Second, because fewer patients presented with elevated HBsAg, the baseline and changing characteristics of liver function, blood tests, CRP, IL-6, and lymphocyte subsets were not reflected in this group of patients. Large prospective clinical trials might elaborate the association between CRP, IL-6, lymphocyte subsets, liver function, blood routine, and HBsAg increase, as well as lead to the discovery of other markers.

## Data Availability Statement

The original contributions presented in the study are included in the article/**Supplementary Material**. Further inquiries can be directed to the corresponding authors.

## Ethics Statement

The studies involving human participants were reviewed and approved by Chinese Ethics Committee of Registering Clinical Trials. The patients/participants provided their written informed consent to participate in this study.

## Author Contributions

SP, YY, F-SW, FM, and MS conceived the study. SP and YY wrote the manuscript. SP, YY, and SW collected data and performed the data analysis. SW, BT, YS, XL, NS, and YZ participated in the clinical treatment. QQ and JL supervised the clinical treatment. F-SW, FM, MS and J-YZ directed the writing and revision of the manuscript. All authors contributed to the article and approved the submitted version.

## Funding

This work was supported by grants from the National Natural Science Foundation of China (82070617); the Innovative Research Group Project of the National Natural Science Foundation of China(81721002); Beijing Municipal Science and Technology Commission (Z201100005520047) and National Key Research and Development Program of China (2019YFC0840704).

## Conflict of Interest

The authors declare that the research was conducted in the absence of any commercial or financial relationships that could be construed as a potential conflict of interest.

The reviewer XZ declared a shared parent affiliation with the author NS to the handling editor at the time of review.

## Publisher’s Note

All claims expressed in this article are solely those of the authors and do not necessarily represent those of their affiliated organizations, or those of the publisher, the editors and the reviewers. Any product that may be evaluated in this article, or claim that may be made by its manufacturer, is not guaranteed or endorsed by the publisher.
