# Supplementary material for: Correlation of HBV DNA and Hepatitis B Surface Antigen Levels With Tumor Response, Liver Function and Immunological Indicators in Liver Cancer Patients With HBV Infection Undergoing PD-1 Inhibition Combinational Therapy
Source: Front Immunol. 2022 May 25;13:892618. doi: 10.3389/fimmu.2022.892618 (PMC9195870; doi:10.3389/fimmu.2022.892618)
Supplement: Supplementary file 1 [file Table_1.docx]

**Table S1** Characteristics of 11 patients with elevated levels of HBV DNA

|  | **Patient 1** | **Patient 2** | **Patient 3** | **Patient 4** | **Patient 5** | **Patient 6** | **Patient 7** | **Patient 8** | **Patient 9** | **Patient 10** | **Patient 11** |
| --- | --- | --- | --- | --- | --- | --- | --- | --- | --- | --- | --- |
| **Age** | 48 | 64 | 48 | 58 | 42 | 62 | 46 | 38 | 45 | 53 | 75 |
| **Gender(M/F)** | M | F | M | M | M | M | M | M | M | M | M |
| **Diagnosis** | HBV-HCC | HBV-HCC | HBV-HCC | HBV-HCC | HBV/ICC | HBV-HCC | HBV-HCC | HBV-HCC | HBV-HCC | HBV-HCC | HBV-HCC |
| **Child-Pugh** | B/7 | B/7 | B/8 | A/6 | B/8 | A/6 | A/6 | A/6 | A/6 | B/7 | A/6 |
| **Previous antiviral therapy** | + | + | + | + | + | + | + | + | + | + | - |
| **Prior anti-tumor therapy** | TACE, Sorafenib | - | TACE, Sorafenib | - | TACE, Sorafenib | TACE, Lenvatinib | Lenvatinib | TACE, Lenvatinib | Hepatectomy, TACE, Sorafenib | - | - |
| **BCLC** | B | C(PVTT) | C(M) | C(M) | C(M) | B | C(M) | C(PVTT) | C(PVTT) | C(M) | C(M) |
| **Antiviral therapy** | TDF+ETV | ETV | ETV | ETV | TDF+ETV | ETV | TDF | ETV | TDF | ETV | - |
| **PD-1 ICIs** | Sintilimab | Sintilimab | Sintilimab | Sintilimab | Sintilimab | Sintilimab | Sintilimab | Sintilimab | Sintilimab | Camrelizumab | Sintilimab |
| **Targeted therapy** | Sorafenib | Lenvatinib | Lenvatinib | Lenvatinib | Lenvatinib | Lenvatinib | Lenvatinib | Lenvatinib | Lenvatinib | Lenvatinib | Lenvatinib |
| **Interventional therapy** | - | - | - | - | - | - | - | - | + | - | - |
| **HBV-DNA**  **(log_10_IU/ml)** |  |  |  |  |  |  |  |  |  |  |  |
| **baseline** | 2.7 | 1.3 | 1.3 | 1.74 | 4.17 | 1.35 | 1.57 | 3.52 | 1.3 | 1.3 | 2.34 |
| **12 weeks** | 2.61 | 3.08 | 1.3 | 2.4 | 4.69 | 2.24 | 2.36 | 4.71 | 1.3 | / | 2.46 |
| **24 weeks** | 3.39 | / | 2.16 | / | / | 2.61 | 1.3 | 1 | 2.74 | 2.97 | / |
| **HBsAg(log_10_IU/ml)** |  |  |  |  |  |  |  |  |  |  |  |
| **baseline** | 2.95 | 2.31 | -0.74 | 2.39 | 3.019 | 3.28 | 4.28 | 3.25 | 2.83 | 3.02 | -1.3 |
| **12 weeks** | 2.97 | 2.14 | -0.67 | 0.699 | 3.15 | 3.08 | / | / | 2.8 | / | -1.3 |
| **24 weeks** | 3.49 | / | 0.11 | / | / | 3.23 | 4.08 | / | 2.7 | 3.02 | / |
| **ALT/AST(U/L)** |  |  |  |  |  |  |  |  |  |  |  |
| **baseline** | 36/64 | 45/78 | 65/108 | 64/101 | 32/127 | 42/47 | 79/55 | 54/110 | 20/32 | 22/25 | 39/56 |
| **12 weeks** | 24/122 | 30/36 | 19/34 | 27/39 | 43/382 | 57/40 | 195/157 | 44/148 | 22/35 | / | 56/70 |
| **24 weeks** | 33/175 | / | 82/79 | 37/87 | / | 59/67 | 63/73 | / | 31/32 | 75/153 | / |
| **Adverse events** | / | AIH(grade3/4) | Fever; hypothyroidism; | / | / | Rash; fatigue; nausea | / | Diarrhea; pneumonia; TBIL rised(grade3/4) | Fatigue; hypertension; fever | / | / |
| **Tumor responses** | PD | PD | PD | PD | PD | PD | PD | PD | PR | PD | SD |
| **Survival status** | death | death | death | survival | death | survival | death | death | lost | death | lost |

**Abbreviations**: **BCLC**, Barcelona Clinic Liver Cancer; **ICIs**, immune checkpoint inhibitors; **M**, metastasis; **PVTT**, portal vein tumor thrombus; **TACE**, transcatheter arterial chemoembolization; +: positive; -: negative; /: no data.
