# Supplementary material for: Correlation of HBV DNA and Hepatitis B Surface Antigen Levels With Tumor Response, Liver Function and Immunological Indicators in Liver Cancer Patients With HBV Infection Undergoing PD-1 Inhibition Combinational Therapy
Source: Front Immunol. 2022 May 25;13:892618. doi: 10.3389/fimmu.2022.892618 (PMC9195870; doi:10.3389/fimmu.2022.892618)
Supplement: Supplementary file 2 [file Table_2.docx]

**Table S2** Characteristics of 8 patients with elevated levels of HBsAg

|  | **Patient 1** | **Patient 2** | **Patient 3** | **Patient 4** | **Patient 5** | **Patient 6** | **Patient 7** | **Patient 8** |
| --- | --- | --- | --- | --- | --- | --- | --- | --- |
| **Age** | 48 | 48 | 42 | 65 | 55 | 49 | 54 | 63 |
| **Gender(M/F)** | M | M | M | M | M | M | M | M |
| **Diagnosis** | HBV-HCC | HBV-HCC | HBV/iCC | HBV-HCC | HBV-HCC | HBV-HCC | HBV-HCC | HBV-HCC |
| **Child-Pugh** | B/7 | B/8 | B/8 | A/6 | A/6 | A/6 | B/7 | A/5 |
| **Previous antiviral therapy** | + | + | + | + | - | - | + | + |
| **Prior anti-tumor therapy** | TACE, Sorafenib | TACE, Sorafenib | TACE, Sorafenib | - | - | - | TACE, Sorafenib | TACE, Sorafenib, radioterapy |
| **BCLC** | B | C(M) | C(M) | C(PVTT) | C(M) | B | C(PVTT) | C(M) |
| **Antiviral therapy** | TDF+ETV | ETV | TDF+ETV | TDF+ETV | ETV | ETV | ETV | ADV |
| **Immunity therapy** | Sintilimab | Sintilimab | Sintilimab | Toripalimab | Sintilimab | Sintilimab | Sintilimab | Camrelizumab |
| **Targeted therapy** | Sorafenib | lenvatinib | lenvatinib | lenvatinib | lenvatinib | lenvatinib | lenvatinib | lenvatinib |
| **TACE treatment** | - | - | - | - | - | + |  |  |
| **Interventional therapy** | - | - | - | - | - | - | + | - |
| **HBV-DNA(log_10_IU/ml)** |  |  |  |  |  |  |  |  |
| **baseline** | 2.7 | 1.3 | 4.17 | 3.87 | 6.07 | 6.19 | 1.3 | 2.08 |
| **12 weeks** | 2.61 | 1.3 | 4.69 | 1.30 | 2.08 | 2.34 | 1.3 | 1.94 |
| **24 weeks** | 3.39 | 2.16 | / | 1.30 | / | 1.33 | 2.74 | 1.30 |
| **HBsAg(log_10_IU/ml)** |  |  |  |  |  |  |  |  |
| **baseline** | 2.95 | -0.74 | 3.019 | 1.734 | 3.66 | 3.27 | 2.83 | 2.42 |
| **12 weeks** | 2.97 | -0.67 | 3.15 | 1.737 | 3.67 | / | 2.8 | / |
| **24 weeks** | 3.49 | 0.11 | / | / | / | 3.51 | 2.7 | 2.70 |
| **ALT/AST(U/L)** |  |  |  |  |  |  |  |  |
| **baseline** | 36/64 | 65/108 | 32/127 | 12/108 | 49/62 | 13/22 | 20/32 | 31/52 |
| **12 weeks** | 24/122 | 19/34 | 43/382 | 20/45 | 32/62 | 58/118 | 22/35 | 27/58 |
| **24 weeks** | 33/175 | 82/79 | / | 22/74 | / | 40/53 | 31/32 | 34/60 |
| **Adverse events** | / | Fever, hypothyroidism | / | / | / | / | / | / |
| **Tumor responses** | PD | PD | PD | PD | SD | SD | SD | SD |
| **survival status** | death | death | death | death | lost | survival | survival | death |

**Abbreviations**: **BCLC**, Barcelona Clinic Liver Cancer; **ICIs**, immune checkpoint inhibitors; **M**, metastasis; **PVTT**, portal vein tumor thrombus; **TACE**, transcatheter arterial chemoembolization; +: positive; -: negative; /: no data.
